# Fibrosis Evaluation by Transient Elastography in Patients With Long-Term Sustained HCV Clearance

**DOI:** 10.5812/hepatmon.7176

**Published:** 2013-05-11

**Authors:** Vincenza Calvaruso, Vito Di Marco, Donatella Ferraro, Salvatore Petta, Anna Calì, Maria Grazia Bavetta, Elisabetta Conte, Piero Luigi Almasio

**Affiliations:** 1 Gastroenterologia and Epatologia, Di.Bi.M.I.S., University of Palermo, Palermo, Italy; 2 Dipartimento di Igiene G.D’Alessandro, University of Palermo, Palermo, Italy

**Keywords:** Liver Cirrhosis, Elasticity Imaging Techniques, Insulin Resistance

## Abstract

**Background:**

Reversibility of advanced fibrosis after HCV-clearance is an important goal of therapy.

**Objectives:**

Measuring liver stiffness (LS) by transient elastography (TE) might be helpful in this setting.

**Patients and Methods:**

We evaluated 104 patients with biopsy-proven chronic hepatitis C (CHC) and sustained virological response (SVR) after Peg-Interferon (IFN) plus ribavirin since at least 18 months. HCV-eradication was confirmed searching for serum HCV-RNA (TMA® sensitivity > 5-10 IU/ml). Data from literature reported the best LS cut-off values for different stages of liver fibrosis were 7.1 kPa for Metavir stage 2 (F2), 9.5 kPa for F3 and 12.5 for cirrhosis (F4).

**Results:**

TE was not reliable in four SVR obese patients. Metavir-stage of biopsy was F0-1 in 28, F2 in 47, F3 in 17 and F4 in eight patients. The median interval elapsed since achieving SVR was 36 months (range: 18-77, SD¬¬:18). Stratifying patients according to the histological stage assessed before treatment, a clear-cut gradient of LS values was observed from F0-1: median: 3.8 kPa (range: 3.5-4.9) to F2: 4.6 kPa (3.8-6.0), F3: 6.2 kPa (4.8-8.6) and F4: 8.4 kPa (6.2-9.2) (P = 0.001). Overall, 86 patients had lower values of LS than the expected LS values according to Metavir-stage. At multivariate logistic analysis γ-GT and histological steatosis were independently associated with persistence of higher values of LS.

**Conclusion:**

Long term responders to IFN-based therapies have lower LS values than those who are untreated and still viraemic. High levels of γ-GT and liver steatosis, all markers of insulin resistance, may hamper reduction of liver stiffness after HCV-clearance.

## 1. Background

Evidence of either fibrotic or cirrhotic regression has now been documented in the entire spectrum of chronic liver diseases, including autoimmune hepatitis (1), biliary obstruction (2), iron overload (3), Nonalcoholic Steatohepatitis (NASH) (4, 5), and viral hepatitis (6-10). In patients with chronic hepatitis C, IFN-based treatment has been reported to be responsible for the regression of liver fibrosis in patients with sustained virological response (SVR) (10, 11). Diminishing fibrosis is an indication of improved long-term prognosis induced by treatment. The detailed histological analysis of the liver biopsies showing almost complete disappearance of inflammation, concomitant with lessening fibrosis scores in patients with SVR, indicates cure of the chronic HCV infection. Thus, not only did fibrosis not progress, but a clear-cut improvement in the fibrosis score in the follow-up biopsy, as compared to that taken before treatment, was seen in long-term responders, in contrast to the findings in patients who failed to achieve a SVR. Nowadays liver biopsy is still considered the gold standard for staging fibrosis. However, liver biopsy is no more considered as a perfect methodology because of the invasive nature of the procedure, sampling error and inter-observer variability (12, 13). Therefore, further alternative reliable strategies are needed for assessment of the hepatic status in patients with chronic liver diseases. In the last years, LS measurement by TE has been proposed as a rapid and non-invasive tool to evaluate the stage of chronic liver diseases (CLD). A strong association of LS with the stage of liver disease has been convincingly demonstrated in patients with chronic hepatitis (14, 15) and a recent study has confirmed that TE is highly reproducible and characterized by an excellent inter and intra-observer agreement (16). For these features, TE may be helpful in assessing liver fibrosis in patients who achieved a SVR.

## 2. Objectives

- To assess the capability of TE to evaluate the improvement of liver damage during long-term follow-up of patients clearing HCV after antiviral therapy with Peg-IFN plus ribavirin.

- To identify the variables associated with the persistence of liver damage evaluated by TE in patients which achieved a SVR.

## 3. Patients and Methods

All patients with virological and histological diagnosis of CHC visited at Gastroenterology and Hepatology Unit of the University Hospital, Palermo from January 2004 to December 2007 and successfully treated with Peg-IFN plus ribavirin were enrolled in the study. To confirm SVR, HCV genome was investigated by COBAS Amplicor HCV Monitor, version 2.0; Roche or COBAS Amplicor HCV 2.0, sensitivity 50 IU/mL. Patients with HBV/HIV co-infection, alcohol abuse were excluded from the study. Biochemical and virological tests, and TE were always performed on the same day. Informed consent was obtained from each patient included in the study and the study protocol conforms to the ethical guidelines of the 1975 Declaration of Helsinki as reflected in a priori approval by the institution's human research committee. None of the patients declined to give consent.

### 3.1. Clinical and Laboratory Assessment

Clinical and anthropometric data were collected at the time of TE. BMI was calculated on the basis of weight in kilograms and height (in meters). A 12-hour overnight fasting blood sample was drawn at the time of biopsy and of TE to determine serum levels of ALT, γ-glutamyl transpeptidase (γ-GT), total cholesterol, HDL and LDL-cholesterol, triglycerides, ferritin, plasma glucose concentration, and platelet count. All patients were tested at the time of biopsy for HCV-RNA by qualitative PCR (Cobas Amplicor HCV Test version 2.0; limit of detection: 50 IU ⁄ mL). HCV RNA positive samples were quantified by Versant HCV RNA 3.0 bDNA (Bayer Co., Tarrytown, NY, USA) expressed in IU/mL. Liver stiffness measurements were performed by two expert physicians (VC, AC) using TE (FibroScan, Echosens, Paris, France). Details of the technical background and examination procedure have been previously described (17). Up to 10 successful measurements were performed on each patient. Success rate was calculated as the ratio of the number of successful measurements over the total number of acquisitions. Only those obtained with at least a success rate of at least 60% were considered reliable. The results are expressed in kilopascal (kPa). Median value of the successful measurements was the estimated value of liver stiffness. We assessed interobserver variability between the two operators using 15 cases and 15 controls (15% of both cohorts) selected at random. Each physician was unaware of the assessments of the other one. To evaluate if the LS results were lower than expected according stage of fibrosis we used the previous published cut offs (F2: 7.1 kPa; F3: 9.5 kPa and F4: 12.5) (14).

### 3.2. Liver Histology

Liver biopsies performed before antiviral treatment was reevaluated by a single pathologist blinded from previous histological, clinical and biochemical results. The liver fibrosis stage was reported using a five-point (F0 –F4) scale: Stage F0 indicated absence of fibrosis; F1 expressed portal fibrosis without septa; F2 was equal to portal fibrosis with a few septa; F3 indicated numerous septa without cirrhosis; and F4 was equivalent to liver cirrhosis. The percentage of hepatocytes containing macrovescicular fat was determined for each 10x field. An average percentage of steatosis was then determined for the entire specimen. Steatosis was assessed as the percentage of hepatocytes containing fat droplets (minimum 5%), and evaluated as a continuous variable. Steatosis was classified as: mild 5%-30% or moderate-severe ≥ 30%.

### 3.3. Statistical Analysis

Data were collected with a predefined pro-forma. Continuous variables were summarized as mean and standard deviation and categorical variables as frequency and percentage. Differences in the means were evaluated by an unpaired Student t-test and the Chi-squared test was applied to categorical variables. Spearman correlation analysis was used to assess the relationships between biochemical, virological, histological variables and TE. Significant variables on univariate analysis (P < 0.05) were included in multivariate model. Multiple logistic regression models were used to assess the demographic, virological, metabolic, instrumental and histological characteristics of patients associated to the persistence of high value of liver stiffness. All statistical analyses were performed with SPSS 17.0 (SPSS, Chicago, IL).

## 4. Results

One hundred and four consecutive patients with chronic hepatitis C diagnosed between January 2004 to December 2006 at Gastroenterology and Hepatology Unit of the University Hospital of Palermo and treated with Peg-IFN plus Ribavirin obtaining a SVR was enrolled in the study. Four patients had an unreliable TE due to obesity (BMI > 30). Table 1 shows the demographic, clinical and histological characteristics of SVR cohort. There were 54 male patients and mean age was 56.2 ± 11.8 years. Mean value of BMI was 27.8 ± 4.0 Kg/h2. Mean total cholesterol and triglycerides values were 192.5 ± 36.5 mg/dl and 120.1 ± 56.7 mg/dl respectively. To assess the presence of insulin resistance we evaluated the triglycerides/glucose index (TyG). Mean value of TyG was 4.62 ± 0.24. As expected, mean values of aminotransferases, γ-GT, bilirubin and platelets count were in normal ranges. The distribution of Metavir histological stages at liver biopsy performed before antiviral treatment in SVR patients was the following: stage F0-F1 was reported in 28 patients (28%), F2 in 47 (47%), and F3 in 17 (17%). Finally, 8 patients (8%) were classified as having cirrhosis (Metavir stage = 4). Histological steatosis was found in 24% of patients.

**Table 1.** Demographic, Clinical and Histological Features of CHC Patients With Sustained Virological Response (SVR)

	SVR patients (n = 100)
**Males, No. (%)**	54 (54.0)
**Age, y, mean ± SD**	56.2 ± 11.8
**Total Bilirubin, mg/dl, mean ± SD**	0.8 ± 0.3
**AST, IU/l mean ± SD**	19.8 ± 6.8
**ALT, IU/l mean ± SD**	20.5 ± 10.3
**Platelet count x 10^3^/mmc, mean ± SD**	220.3 ± 67.2
**γ-GT, IU/l, mean ± SD**	21.4 ± 14.6
**Total Cholesterol, mg/dl, mean ± SD**	192.5 ± 36.5
**Triglycerides, mg/dl, mean ± SD**	120.1 ± 56.7
**TyG test, mean ± SD**	4.62 ± 0.24
**Diabetes, No. (%)**	9 (9.0)
**Body mass index (mean ± SD)**	27.8 ± 4.0
**Genotype 1/4**	72
**Genotype 2/3**	28
**HCV-RNA, UI/mL, mean ± SD**	_
**Stage of fibrosis (Metavir score), No. (%)**	
0-1	28 (28.0)
2	47 (47.0)
3	17 (17.0)
4	8 (8.0)
**Grade of inflammation (Metavir score), No. (%)**	
1	28 (28.0)
2	46 (46.0)
3	26 (26.0)
**Histologicalsteatosis**	24 (24.0)

### 4.1. TE Data Analysis

Using the LS cut-offs values for different stage of liver fibrosis reported in the Castera et al study (14) we found that in 86 patients (86%), TE results were lower respect the referral cut-offs values defined for the different Metavir stages. In particular, a TE value lower than 7.1 kPa was observed in 66 of the 76 (86.8%) patients with Metavir stage ≤ 2, 13 out of 17 SVR patients (76.5%) who had severe fibrosis (Metavir stage 3) had a TE value lower than 9.5 kPa and finally, in all eight patients with cirrhosis the TE value in the follow up was lower than 12.5 kPa (100%).

### 4.2. Variables Related to TE in SVR Patients

By univariate linear regression analysis TE was significantly correlated with γ-GT, platelets, Metavir stage and BMI. The variables independently associated with TE as continuous variables were only Metavir stage and BMI by multivariate linear regression analysis (Table 2).

**Table 2.** Spearman Correlation Analysis of Liver Stiffness With Clinical Variables in 100 CHC Patients With SVR

	Univariate Analysis		Multivariate Analysis		
	Correlation coefficient	P value	β^a^	S.E.	P value
**Age**	0.087	0.4			
**AST^a^**	0.108	0.3			
**ALT^a^**	0.182	0.1			
**γ-GT^a^**	0.400	< 0.001	0.026	0.017	0.1
**Platelet count**	-0.315	0.014	-0.004	0.004	0.3
**Total Cholesterol**	0.002	0.9			
**Tyg^a^**	0.181	0.1			
**Stage of Fibrosis (Metavir Score)**	0.475	< 0.001	0.943	0.302	0.003
**Body mass index**	0.286	0.010	0.179	0.073	0.018

^a^Abbreviations: AST, aspartate aminotransferase; ALT, alanine aminotransferase, β, beta coefficient; γ-GT: gamma-glutamyl-transpeptidase; TyG: triglycerides/glucose index

### 4.3. Variables Associated With Persistence of High TE Values

Since in 14 patients we have observed a persistently high value of TE despite the SVR, we analyzed if some clinical variables may predict the persistence of high value of TE in these patients. Univariate and multivariate logistic regression analyses were performed to identify the variables associated to the persistence of high values of TE in patients with SVR. At univariate analysis the absence of reduction of TE was associated with BMI, γGT and histological steatosis. Age, diagnosis of diabetes and Metavir stage were marginally associated to high TE values at univariate analysis and included in the multivariate model. Histological steatosis (OR 2.80, 95%CI 1.000 – 12.52; P = 0.050) and γ-GT (1.06 95%CI 1.01-1.12; P = 0.014), remain the unique variables independently associated to permanence of high TE at multivariate analysis (Table 3). Excluding steatosis and Metavir stage from the model, since they could be verified only by biopsy, a model was obtained that included γ-GT (OR 1.07; 95%CI 1.01 - 1.13; P = 0.041) and BMI (OR 1.22; 95%CI 1.01 - 1.50; P =0.045) as indirect predictors of higher TE values (Table 4).

**Table 3.** Logistic Regression Analysis of Variables Associated to Persistently High Value of Liver Stiffness (LS) in 100 Patients With CHC and SVR

Variable	Not improved LS, (n=14)	Improved LS^a^, (n=86)	P value	O.R. (95% C.I.)	P value
**Male gender, No. (%)**	9 (64.3)	43 (50.0)	0.41		
**Age, y, Mean ± SD**	60.8 ± 8.3	55.6 ± 12.3	0.07	1.02 (0.95-1.09)	0.704
**AST^a^, Mean ± SD**	17.7 ± 4.4	20.1 ± 7.2	0.33		
**ALT,^a^Mean ± SD**	19.6 ± 7.4	20.3 ± 10.7	0.79		
**γ GT^a^, Mean ± SD**	37.2 ± 23.7	21.9 ± 14.3	0.04	1.06 (1.01-1.12)	0.014
**Platelet count, Mean ± SD**	205.0 ± 33.6	217.9 ± 73.8	0.65		
**Total Cholesterol, Mean ± SD**	193.4 ± 38.5	192.5 ± 34.9	0.91		
**Insulin resistance^b^, Mean ± SD**	8 (57.1%)	39 (45.3%)	0.28		
**Body mass index, Mean ± SD**	29.8 ± 5.4	27.1 ± 3.4	0.03	1.19 (0.96-1.47)	0.101
**Diabetes, No. (%)**	3 (21.4%)	4 (4.6%)	0.08	4.04 (0.43-38.04)	0.223
**Stage of fibrosis (Metavir score), No. (%)**					
0-1	1 (7.1)	27 (31.4)	0.052	1.25 (0.54-2.92)	0.605
2	9 (64.3)	38 (44.2)			
3	4 (28.6)	13 (15.1)			
4	0	8 (9.3)			
**Steatosis**	6 (42.8)	16 (18.6)	0.03	2.80 (1.00-12.52)	0.050

^a^Abbreviations: AST, aspartate aminotransferase; ALT, alanine aminotransferase; γ-GT, gamma-glutamyl-transpeptidase; LS, Liver Stiffness

^b^(TyG ≥ 4.6)

**Table 4.** Logistic Regression Analysis of Variables Associated to Persistently High Value of Liver Stiffness in CHC Patients With SVR, Histological Variables Were Excluded From the Model

Variable	Not improved LS^a^, (n = 13)	Improved LS, (n = 86)	P value	O.R. (95% C.I.)	P value
**Male gender, No. (%)**	9 (64.3)	43 (50.0)	0.41		
**Age, y, Mean ± SD**	60.8 ± 8.3	55.6 ± 12.3	0.07	1.01 (0.94-1.09)	0.818
**AST^a^, Mean ± SD**	17.7 ± 4.4	20.1 ± 7.2	0.33		
**ALT^a^, Mean ± SD**	19.6 ± 7.4	20.3 ± 10.7	0.79		
**γ-GT^a^, Mean ± SD**	37.2 ± 23.7	21.9 ± 14.3	0.04	1.07 (1.01-1.13)	0.041
**Platelet count, Mean ± SD**	205.0 ± 33.6	217.9 ± 73.8	0.65		
**Total cholesterol, Mean ± SD**	193.4 ± 38.5	192.5 ± 34.9	0.91		
**Insulin resistance^b^, No. (%)**	8 (57.1)	39 (45.3)	0.28		
**Body mass index, Mean ± SD**	29.8 ± 5.4	27.1 ± 3.4	0.03	1.22 (1.01-1.50)	0.045
**Diabetes, No. (%)**	3 (21.4)	4 (4.6)	0.08	3.40 (0.42-27.82)	0.254

^a^Abbreviations: AST, aspartate aminotransferase; ALT, alanine aminotransferase; γ-GT, gamma-glutamyl-transpeptidase; LS, Liver Stiffness

^b^TyG ≥ 4.6

## 5. Discussion

TE is a non-invasive device designed to predict liver fibrosis. This methodology is currently subject to extensive validation in cross-sectional and longitudinal studies but, currently, its role in the evaluation of regression of liver fibrosis has not been extensively studied. The present study have shown a significant reduction of the mean values of LS in the SVR patients respect the best TE cut off values reported in literature in patients infected by HCV. This confirmed the results observed by Ogawa et al. (18) who demonstrated that the liver elasticity of SVR patients markedly improved over time. However, the reasons of this phenomenon are not completely understood. In fact, we already know that TE measurement is strongly influenced by liver inflammation (19-21), thus the reduction of liver stiffness value could be entirely correlated to the decrease of activity in the liver. On the other side, many previous reports have shown that IFN treatment achieves biochemical and histological improvement with viral suppression in patients with CHC (9, 11, 22-24). Several potential mechanisms have been hypothesized for the anti-fibrotic effect of IFN, including that IFN alpha can directly reduce fibrogenesis (9, 10). Thus TE in this cohort detected a reduction of the fibrosis amount in the liver and taking into account that liver biopsy has not been recommended in patients who achieve SVR, it has a useful role in the assessment of the correlation between liver stiffness and liver fibrosis in the no longer active viral disease. Further studies should be performed with the aim to asses if in cirrhotic patients undergoing antiviral therapy TE may have a role in a noninvasive prognostic evaluation of advanced liver disease and to evaluate any clinical differences between patients who achieve the reduction of liver stiffness in comparison with those without SVR. Another aim of our study was evaluate the demographic, clinical and histological variable associated to a persistence of high TE value in patients with SVR after IFN based treatment. It is well known from literature that Insulin Resistance (IR), a feature exceedingly common in patients with CHC (25-28), particularly in genotype 1 (G1) infection (26), can be considered a putative candidate for influencing LSM variations. In this setting, IR has been systematically associated not only with liver steatosis, but also with severe fibrosis (26-28), portal hypertension (29, 30) and, ultimately, with the mechanisms leading to collagen deposition, vasoconstriction, and regulation of sinusoidal structure (31-34). Therefore, insulin, interfering with all the above cited mechanisms, could influence liver stiffness. Poynard et al. (9) have shown that the factors associated with a regression of liver fibrosis for patients with chronic HCV after antiviral therapy are baseline fibrosis stage, baseline activity, SVR, BMI, age and viral load. In our study we have observed that the by univariate analysis, the 14 SVR patients with persistently higher liver stiffness value had a significantly higher γ-GT and BMI values and an higher prevalence of diabetes and liver steatosis at liver biopsy respect the other 86 patients. Excluding histological steatosis, taking into account that we need on a non-invasive assessment of liver damage in this category of patients, we have found that only γ-GT and BMI were independently associated to high TE results by multivariate analysis, suggesting an important role of metabolic cofactors on the regression of liver fibrosis which can be measured also by TE. This study has some limitations. First, the analysis was carried out in a relatively small number of patients, and it would be interesting to see if these results also hold true in larger groups of patients with CHC who obtain SVR. Second, our study included a cohort of European, non-drinker patients with a low prevalence of obesity and cirrhosis, who were enrolled in a tertiary referral center for liver disease, limiting the broad application of the results. Third, our patients started antiviral therapy when TE was not available. Therefore we used well established TE cutoff values of different fibrosis stages to evaluate the changes of LS values at the end of follow-up. A further methodological limitation could reside in the accuracy of liver biopsy examination for assessing fibrosis, which is universally defined as “gold standard”. However, it is widely known that liver biopsy should be considered a “reference standard”, because of the limitations previously discussed. In conclusion, our results show that transient elastography is a useful tool for the longitudinal assessment of IFN treatment of chronic hepatitis C patients and that in CHC patients who eradicate HCV it may help the clinicians for the assessment of the outcome of these patients and to identify and treat the cofactors.
